# Management of trauma White weapons penetrating Head and Neck in the anesthesia department of the national hospital of Zinder in Niger: About five reported cases

**DOI:** 10.1016/j.amsu.2022.103840

**Published:** 2022-06-03

**Authors:** A. Magagi, M.S. Rabiou, M. Maikassoua, R. Habibou, M.L. Hassan, M.B. Boukari, M.S. Chaibou, H. Daddy

**Affiliations:** a Department of Anaesthesia and Intensive Care of the National Hospital of Zinder, Faculty of Health Sciences, The University of Zinder in Niger, BP, 613, Niger; b Neurosurgery Department of the National Hospital of Zinder, Niger; c Anesthesiaa and Intensive Care Unit of the Maradi's Referral Hospital, Faculty of the Health Sciences, the Dan Dicko Dankoulodo University of Maradi, Niger; d Department of Anaesthesia and Intensive Care of the National Hospital of Niamey, Niger; e Anesthesia and Intensive Care Unit of the Maradi's Referral Hospital, Faculty of Health Sciences, the Dan Dicko Dankoulodo University of Maradi, Niger

**Keywords:** Management, Penetrating injuries, Head, And neck, Knives, Niger

## Abstract

We report five cases of craniocervical trauma with knives. The occurrence circumstances were common to all injuries. The trauma was caused by a knife during a fight or an intentional injury. All the victims were farmers or ranchers. Their average age was 17 years, with extremes of 13 and 22 years. The cause was most often community conflict. Pre-hospital transport was non-medical for all patients. The average admission time was 3 h and the average management time was 4 h. General anesthesia with orotracheal intubation was the anesthetic technique used. The average length of hospital stay was seven days. The prognosis was overall favorable and the patients returned home without any sequels.

## Highlights

Feedback on the management of craniocervical trauma by knives in the anesthesia department of the national hospital of Zinder in Niger:

- Pre-hospital management: Non-medical pre-hospital management causes a delay in the initial treatment of severe trauma
- Epidemiological aspect: severe and frequent trauma to a very young population
- Therapeutic, and prognostic aspects: Multidisciplinary intra-hospital management with good and strict procedures between emergency physicians, anesthesiologists, and surgeons allows for a favorable prognosis

## 1 Introduction

**Image 1:**
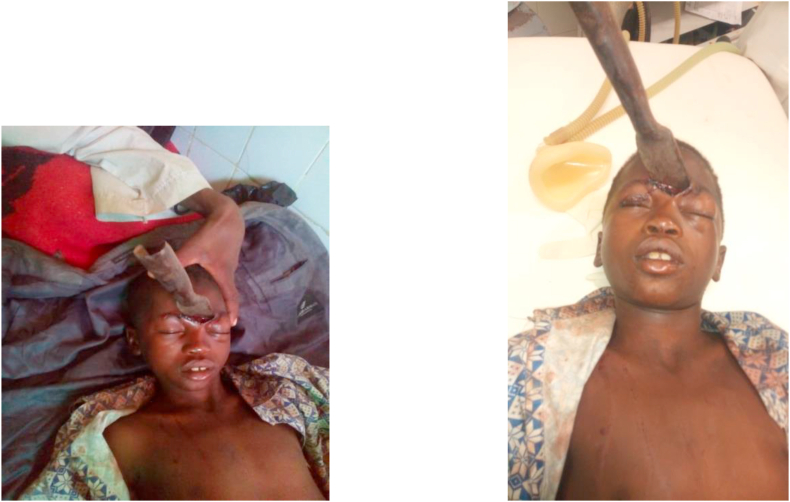
Patient on admission to the emergency department of the national hospital in Zinder.

**Image 2:**
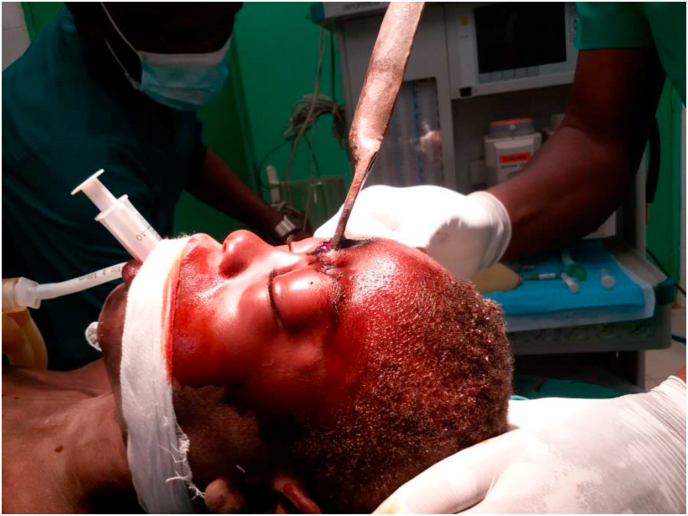
Patient in the operation block with general anesthesia.

**Image 3:**
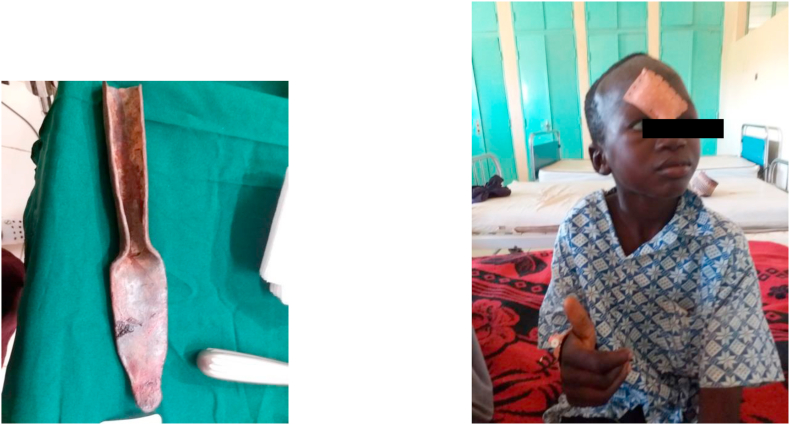
Extracted knife and Patient at discharge.

**Image 4:**
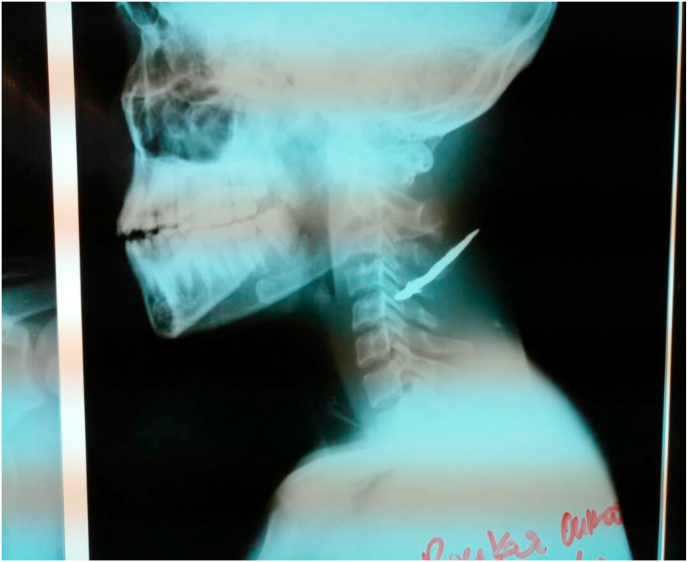
X-ray of the neck of the patient on admission to the emergency room.

**Image 5:**
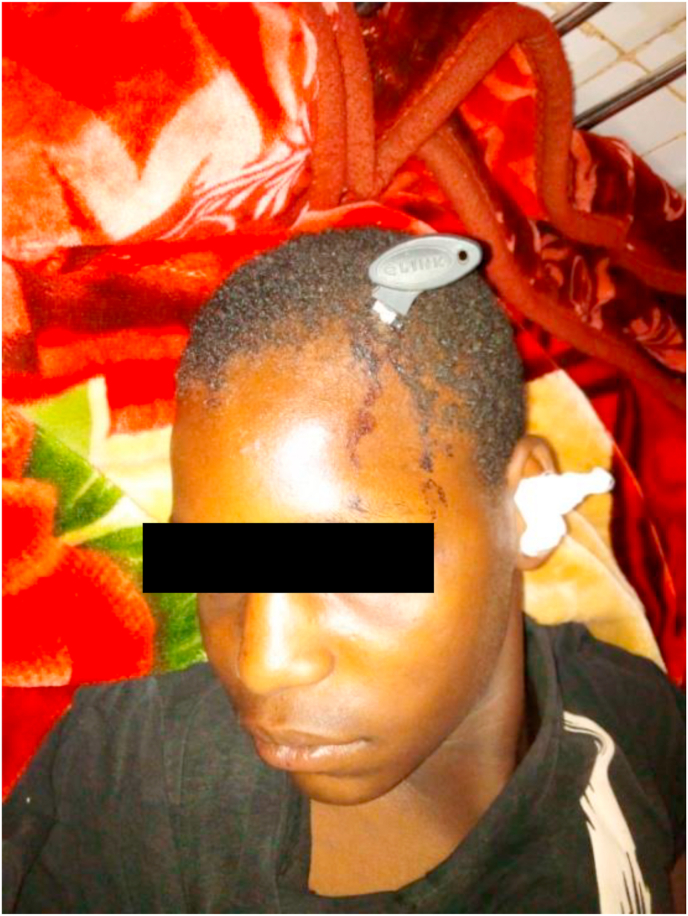
Patient at admission.

**Image 6:**
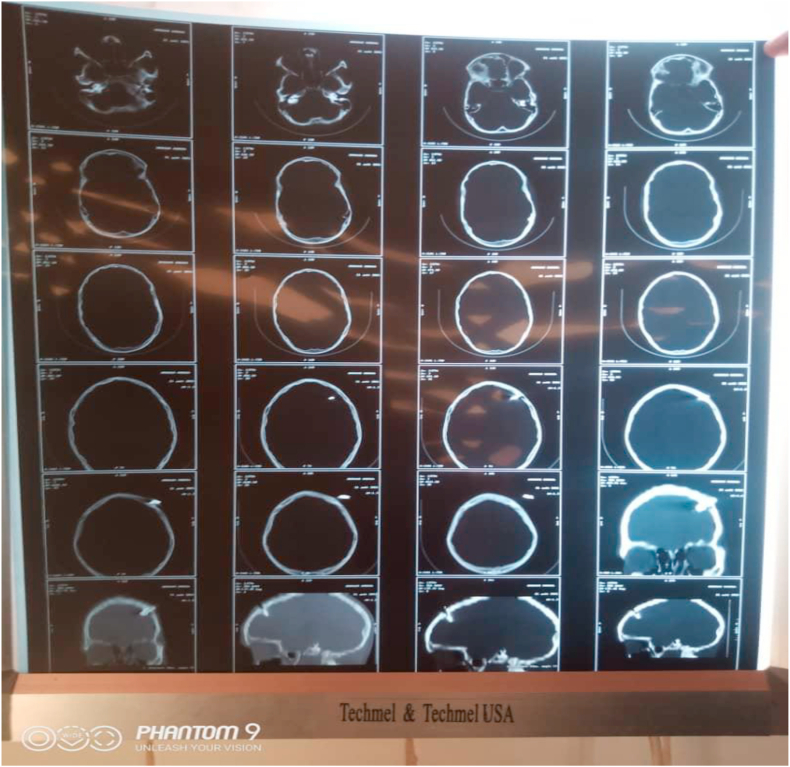
Brain scan.

**Image 7:**
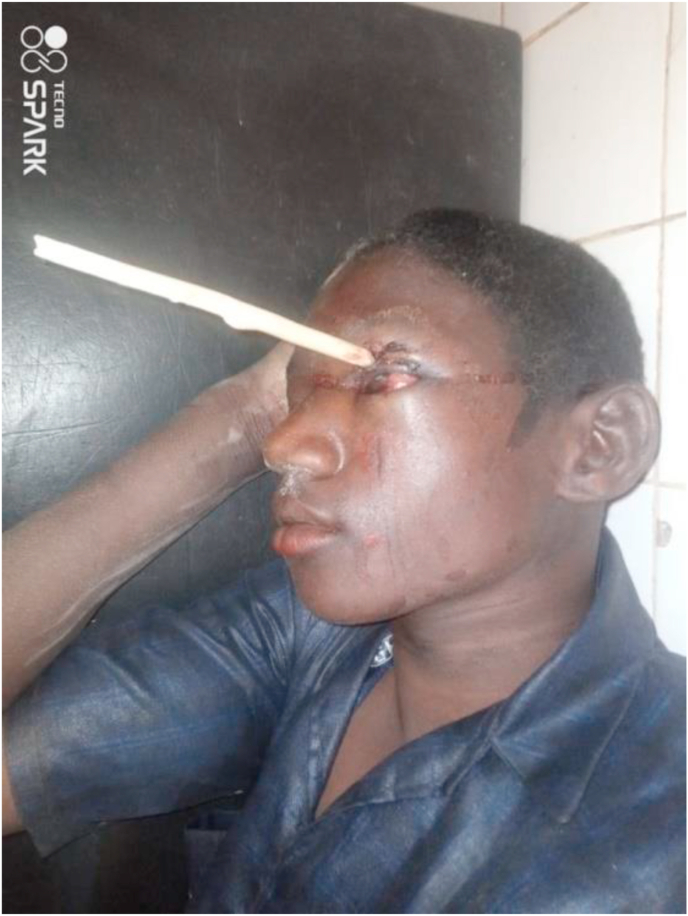
Patient on admission to an emergency department.

**Image 8:**
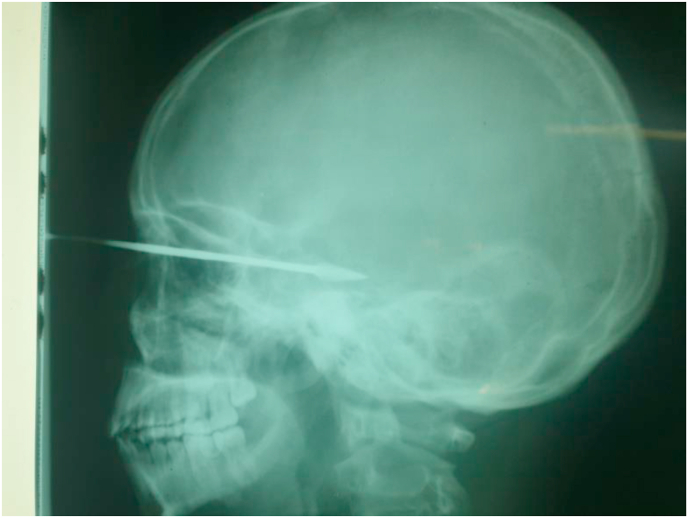
X-ray of the head of the patient at admission.

**Image 9:**
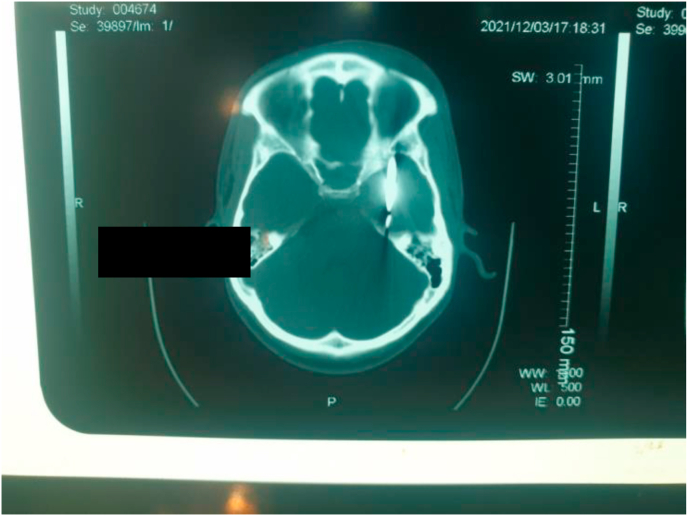
Post-op brain scan.

**Image 10:**
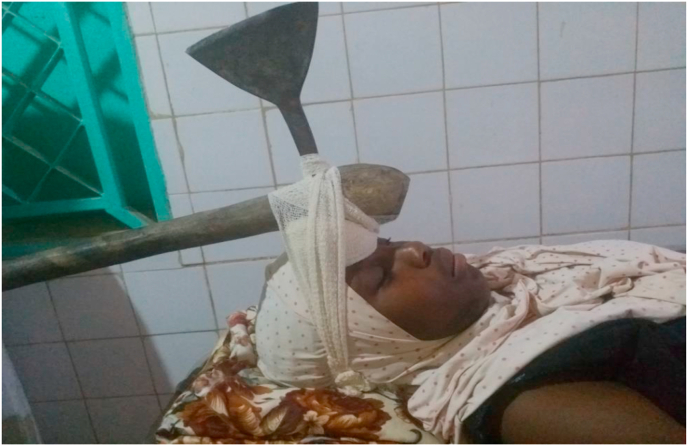
Patient on admission to the Emergency Room.

**Image 12:**
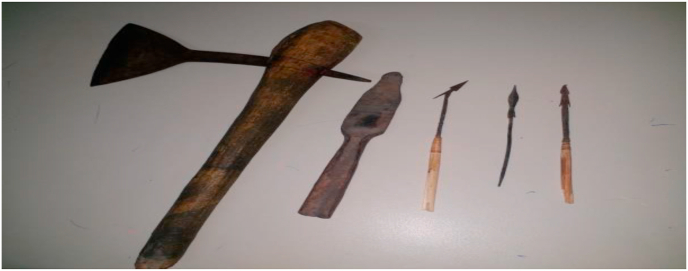
The different bladed weaponsfrom the five patients.

A weapon with a blade or point is a stabbing and/or cutting weapon that does not use the force of an explosion but the force of a man or some mechanism [1]. The blows delivered by a weapon whose propulsion is the human hand have limited energy. The injuries observed are related to the penetrating capacity (stinging, cutting), the size of the penetrating agent, and the anatomical elements concerned by the trajectory of the agent [2]. Bladed weapons (knives, daggers, various blunt objects) produce lesions by the “cutting” effect, which are generally fairly linear. But whatever the weapon or projectile, the seriousness of penetrating injuries is linked to the damage to essential organs, putting the vital prognosis at risk [3]. Penetrating injuries from knives and firearms are a real public health problem in many countries [4]. Their frequency varies according to geographical location: in France, penetrating injuries are not very frequent, representing 10–15% of injuries. In the United States, they account for 70% of injuries, with a majority of firearm wounds [5,6]. In Africa, the highest frequencies are found in South Africa; the Trauma Unit team at Groote Schuur Hospital, Cape Town, counted 400 stab wounds per month [7]. (see Image 1, Image 2, Image 3, Image 4, Image 5, Image 9, Image 7, Image 8, Image 10, Image 12, Image 6).

Traumatic pathologies are increasingly recorded in our health structures. While public road accidents (PVAs) have long been incriminated in the occurrence of PVAs, in our countries we also witness another form of circumstance in the occurrence of traumatic pathology, namely intentional blows and wounds with knives.

Here we reported five cases of craniocervical trauma caused by knives at the national hospital in Zinder in Niger.

## 2 Patients and observations

Our work consists of five case reports and was reported by following the SCARE 2020 criteria [15].

## 3 Clinical case n°1

A 13-year-old patient with no medical or surgical history and up-to-date vaccination was admitted for a head injury caused by a knife. The circumstance of occurrence was intentional injury with a knife during a fight. The patient was reportedly stabbed in the forehead. He was admitted to the emergency department 6 h after the trauma. On admission he was in good general condition, mucous membranes and conjunctiva were well stained; the patient had a Glasgow score of 15 without sensory or motor deficits. The hemodynamic status was stable with regular tachycardia. The lungs were free on auscultation. The rest of the clinical examination was unremarkable. The time to care was 8 h. The patient was classified as ASA II (American Society of Anesthesiologists). After oral and written consent from the child's parents, general anesthesia with orotracheal intubation was the technique used. Anesthetic induction was performed with propofol (4 mg/kg) as hypnotic and suxamethonium (1 mg/kg) as curare used. Crash induction with Sellick maneuver was performed. Anesthetic maintenance was done with halothane and fentanyl. Antibiotic prophylaxis was done with amoxicillin/clavulanic acid (50 mg/kg, intravenous). The anesthetic procedure and recovery were unremarkable, and the postoperative period was simple. The patient was managed by the head surgeon of the department who has fifteen years of experience. The hospitalization lasted 7 days and the patient was discharged without any sequels to his home.

## 4 Clinical case n°2

22-year-old patient admitted for cervical trauma caused by a knife during a fight. He received an arrow in the neck. The admission time was 06 h. On admission, the patient was painful with a visual analog scale (VAS) of 10.

He was 120 pulses/min tachycardia with a mean arterial pressure of 65 mm hg. Stable respiratory function with 98% saturation on room air. The neurological examination noted a Glasgow score of 15/15 and a left hemisphere deficit. The patient was classified as ASA II. After written and oral consent from the patient, the surgery proceeded without complications. The patient was managed by the head surgeon of the department who has fifteen years of experience.The anesthetic technique was general anesthesia with orotracheal intubation. The anesthetic protocol was induction with propofol (4 mg/kg), suxamethonium (1 mg/kg) and the anesthetic maintenance used halothane and fentanyl in doses adapted to the patient's weight. Antibiotic prophylaxis was done with the association amoxicillin/clavulanic acid (50 mg/kg, IV). The tetanus vaccination was up to date. The postoperative period was marked by the absence of left hemisphere deficit. The patient was hospitalized for 12 days and discharged without any sequel.

## 5 Clinical case n°3

A 17-year-old patient with no medical or surgical history was admitted to the emergency department for head trauma caused by a key. The patient was reported to have received a stab wound to the head. The interrogation was revealing that it was the implantation of a key following a fight. The admission time was 4 h. The clinical examination on admission revealed a Glasgow score of 15/15 with no sensory or motor deficit. The clinical examination was normal in terms of hemodynamics and respiratory function. The patient was classified as ASA I. After oral and written consent from the parents, the patient was operating under general anesthesia with orotracheal intubation in crash induction with propofol (4 mg/kg) and suxamethonium (1 mg/kg). Antibiotic prophylaxis was done with the combination of amoxicillin/clavulanic acid (50 mg/kg, IV), and the tetanus vaccination was up to date. Maintenance was done with halothane and fentanyl in doses adapted to the patient's weight. The patient was managed by the head surgeon of the department who has fifteen years of experience. The operation was simple and with no complications. The hospital stay was 5 days and the patient was discharged without any sequels.

## 6 Clinical case n°4

This was an 18-year-old patient with no previous medical or surgical history who was admitted to the emergency department for cranioencephalic trauma. The patient had received an arrow in the left orbit during a fight. The patient was admitted 7 h after the accident. The clinical examination on admission revealed an altered state of consciousness with a Glasgow score of 14/15 without sensory or motor deficits; left monocular blindness with a temporal syndrome. Blood pressure was 130/80 mmHg, and SPO2 was 98% on room air. The anesthetic examination classified the patient as ASA I. After the patient's written and oral consent, the surgery was conducted under general anesthesia with orotracheal intubation and crash induction: propofol (4 mg/kg), suxamethonium (1 mg/kg). Antibiotic prophylaxis was done with the association of amoxicillin/clavulanic acid (50 mg/kg, IV), and the vaccination against tetanus was up to date. The patient was managed by the head surgeon of the department who has fifteen years of experience. The postoperative course was simple. The patient was hospitalized for 5 days and discharged home with a follow-up consultation with an ophthalmologist.

## 7 Clinical case n°5

A 15-year-old patient with no past medical history and up-to-date vaccination was admitted to the emergency department for a head injury caused by a knife. The patient had received the tip of an ax at the frontal region, razing the orbital surface. The admission time was 03 h. The clinical examination was normal. The Glasgow score was 15 with no sensory or motor deficits. The pre-anesthetic visit classified the patient as ASA II. After written and oral consent from the parents, the surgery was conducted without complications under general anesthesia with orotracheal intubation. Crash induction was done with propofol (4 mg/kg), suxamethonium (1 mg/kg), and Sellick maneuver. Maintenance drugs included halothane and fentanyl. Antibiotic prophylaxis was done with the association amoxicillin/clavulanic acid (50 mg/kg, IV). The patient was managed by the head surgeon of the department who has fifteen years of experience. The postoperative course was simple. The patient had returned home after seven (7) days of hospitalization and was declared completely cured.

## 8 Discussion

As a result of socio-economic development, we observe more and more road traffic, which causes traumatic pathologies to multiply. However, in our regions, another form of trauma has recently appeared, namely, blunt force trauma resulting from inter-community conflicts or conflicts between groups of young people.

In our case series, the median age of the patients was 17 years with extremes of 13 and 22 years. All our patients were boys. It appears that stabbing injuries are the prerogative of young adult males. In fact, the circumstances in which they occur are represented by conflictual confrontations or aggressions most often involving men (farmers, herders, traders, gold diggers) [8]. Farmer-herder conflict is recurrent in our context, and almost always results in traumatic or even deadly confrontations. Similar results have been reported by Tintilier and Le Dantec [9,10].

In the majority of the clinical cases observed, the most recorded circumstance of occurrence was inter-community conflict. But sometimes the cause was conflicts or fights between young people. In the case of inter-community conflicts, the explanation lay in disputes between farmers and herders. As for the fights between young people, this was explained by the absence of school education.

The site of the injury is another key factor of severity. Scalp injuries are very bloody. Fractures of the cranial or facial bones are usually evident. The subjacent cerebral lesions will make all the gravity of the craniocerebral lesions. On the face, the noble elements (trigeminal nerve, facial nerve, ocular globe, lacrimal tract, Sténon's canal, facial vascular system) are numerous and superficial and their lesions must be systematically searched for, especially in benign-looking injuries [1]. In our series, the site of the injury was essentially the head in four cases and the neck in one case. The radiology tests had not detected any serious lesions caused by the passage of the knife.

Management consisted of ablation of the material and closure of the injury under general anesthesia in the OR. This could explain the favorable evolution of all our patients.

The severity of knife injuries is correlated to many factors: the type of weapon, the area targeted, and the context of the attack. This information guides the overall management strategy. The weapons used by the attackers of our patients were: knives, keys, arrows, and dabas.

Whatever the mode of transport (Ambulance, Personal Transport, or Fire Brigade), the delays in transporting and accessing the patient to a specialized service, the time of the injury, and the evolution of the patient's condition are to be noted. Among 100 deaths from penetrating trauma, 88% of deaths occurred within the first 30 min and 24% were a direct consequence of head injuries [[11], [12], [13]]. In our series, all patients were admitted by personal means because the national hospital of Zinder does not have an EMS service and the fire brigade service is practically absent from the region. This justified the delay in admission on the one hand and the delays in treatment on the other. The average admission time to the emergency room was 3 h. The authors agreed that the delay in consultation had a significant negative impact on the prognosis of patients. In our series of cases, although the admission time was delayed, the result was positive, with 100% of patients discharged without any sequel. This could be explained by the non-seriousness of the lesions (absence of damage to the large vessels) but also by the quality of the anesthetic and surgical medical care in our series.

All patients had a favorable outcome. No case of death was recorded. This could be explained by: the absence of serious lesions, particularly blood injuries, which are more often the cause of death in pre-hospital than in hospital, and the quality and speed of medical care (anesthetic and surgical care at the National Hospital of Zinder in Niger). Some studies have found that hospital mortality linked to penetrating knife injuries ranges from 2% to 6% in pre-hospital care [14].

## 9 Conclusion

Severe weapon injuries, particularly Head and Neck**,** are becoming more and more frequent in our countries.

Community conflict and fights between young people who do not attend school were the main circumstances. The victims recorded are more likely to be young male adults. The prognosis of patients depends on the severity of the injuries and the quality of the Management.

To improve the quality of care and to reduce the morbidity and mortality of serious weapon injuries in under-developed countries, it is important to underline the importance of the implementation of EMS and SMUR services in the health structures of these countries for medical pre-hospital management.

In order to reduce the incidence of such trauma, it is important that the government should focus on the schooling of young people.

## Authors' contributions

All authors participated in the elaboration of this work and declare that they have read and approved it.

## Sources of funding for your research

The authors declared that this study has received no financial support.

## Ethical approval

Written informed consent was obtained from the patient for publication of this case report and accompanying images. A copy of the written consent is available for review by the Editor-in-Chief of this journal on request.

## Consent

Written informed consent was obtained from the patients for publication of these case reports and accompanying images. A copy of the written consent is available for review by the Editor-in-Chief of this journal on request.

## Author contribution

Habibou Rabiou: The corresponding author and writing the paper. MAGAGI. Amadou: writing the paper. MAIKASSOUA. Mamane: Correction of the paper. Rabiou Sani: Correction of the paper. HASSAN. Maman Lawan: Correction of the paper. DADDY. HADJARA: Correction of the paper. CHAIBOU SANI: Correction of the paper. BOUKARI. BAWA: Correction of the paper.

## Registration of research studies

1. Name of the registry: researchregistry

Unique Identifying number or registration ID: 7819.

2. Hyperlink to your specific registration (must be publicly accessible and will be checked):

## Guarantor

MAGAGI AMADOU .

## Declaration of competing interest

There are no conflicts of interests.
